# Forebrain epileptiform activity is not required for seizure-induced apnea in a mouse model of *Scn8a* epilepsy

**DOI:** 10.3389/fncir.2022.1002013

**Published:** 2022-09-07

**Authors:** Ian C. Wenker, Alexis R. Boscia, Christine Lewis, Anas Tariq, Raquel Miralles, Jessica C. Hanflink, Priyanka Saraf, Manoj K. Patel

**Affiliations:** Department of Anesthesiology, University of Virginia, Charlottesville, VA, United States

**Keywords:** seizure, tonic phase, SUDEP, brainstem, epilepsy, breathing, apnea

## Abstract

Sudden unexpected death in epilepsy (SUDEP) accounts for the deaths of 8–17% of patients with epilepsy. Although the mechanisms of SUDEP are essentially unknown, one proposed mechanism is respiratory arrest initiated by a convulsive seizure. In mice, we have previously observed that extended apnea occurs during the tonic phase of seizures. Although often survived, tonic seizures became fatal when breathing did not immediately recover postictally. We also found that respiratory muscles were tonically contracted during the apnea, suggesting that muscle contraction could be the cause of apnea. In the present study, we tested the hypothesis that pyramidal neurons of the motor cortex drive motor units during the tonic phase, which produces apnea. Mice harboring the patient-derived N1768D point mutation of an *Scn8a* allele were crossed with transgenic mice such that inhibitory Designer Receptors Exclusively Activated by Designer Drugs (DREADD) receptors were selectively expressed in excitatory forebrain neurons. We then triggered audiogenic and hippocampal (HC) stimulated seizures under control conditions and when excitatory forebrain neurons were inhibited with the synthetic ligand Clozapine-*N*-Oxide (CNO). We found that inhibition with CNO was sufficient to increase seizure threshold of HC stimulated, but not audiogenic, seizures. In addition, regardless of seizure type, CNO nearly eliminated epileptiform activity that occurred proximal to the tonic phase; however, the seizure behaviors, notably the tonic phase and concomitant apnea, were unchanged. We interpret these results to indicate that while cortical neurons are likely critical for epileptogenesis and seizure initiation, the behavioral manifestations of tonic seizures are generated by neural circuitry in the mid- and/or hindbrain.

## Introduction

Sudden unexpected death in epilepsy (SUDEP) is defined as the sudden, unexpected, non-traumatic, and non-drowning death of a person with epilepsy for which post-mortem examination does not reveal another cause of death (Nashef et al., 2012). SUDEP is the most common cause of death associated with epilepsy, accounting for up to 17% of all epilepsy-related deaths (Terra et al., 2013), and up to 50% for patients with poorly controlled seizures (Devinsky, 2011; Tolstykh and Cavazos, 2013). Furthermore, amongst all neurological disorders SUDEP is second only to stroke in the number of life years lost (Thurman et al., 2014).

Although SUDEP is likely multi-factorial, peri-ictal apnea and breathing dysfunction are believed to be a primary cause of SUDEP. Apnea and oxygen desaturation have been reported in a large percentage of patients during and after convulsive and non-convulsive seizures (Nashef et al., 1996; Bateman et al., 2008; Lacuey et al., 2018a,b; Vilella et al., 2019), and in nine cases of SUDEP with adequate postictal cardiorespiratory monitoring, terminal apnea occurred prior to terminal asystole (Ryvlin et al., 2013). It is believed that most SUDEP cases occur after generalized convulsive seizures (Dasheiff and Dickinson, 1986; Bird et al., 1997; Nilsson et al., 1999; So et al., 2000; Ryvlin et al., 2013). Thus, mouse models of SUDEP include those in which death occurs immediately after convulsive seizures. Indeed, we and others have found that mouse models of epilepsy that experience early mortality die from seizures that produce apnea. This includes transgenic mice with epilepsy-related mutations such as *Scn8a^N^*^1768*D*^, *Scn8a^R^*^1872*W*^, *Cacna1a^S^*^218*L*^, *Lmx1b^f/f/p^*, and *Scn1a^R^*^1407*X*^, in addition to pharmacological and electrically stimulated models of seizure-induced death (Buchanan et al., 2014; Kim et al., 2018; Jansen et al., 2019; Loonen et al., 2019; Wenker et al., 2021).

Although progress toward understanding mechanisms of SUDEP has been made over the last decade, our understanding of seizure-induced apnea at the level of the CNS is rudimentary. Early studies in rats suggest that seizure spread to the brainstem reticular formation is the cause of apnea, particularly during the tonic phase (Faingold, 2012). In *Scn8a* mutant mice, we have observed tonic contraction of the main breathing muscle, the diaphragm, during the tonic phase (Wenker et al., 2021). This tonic contraction of the diaphragm is sufficient to produce the concomitant apnea; thus, determining which neural circuitry is stimulated by seizure activity to produce apnea is of critical significance. The final output of the central nervous system that controls the musculature are upper motor neurons, which reside in the primary motor cortex, as well as several subcortical regions (Purves et al., 2018). While hypersynchronous activity of cortical neurons, including those of the motor cortex, is a hallmark of convulsive seizures, it is unclear whether the activity of these neurons drive the tonic phase and concurrent apnea.

In the present study, we tested the hypothesis that while excitatory forebrain neurons are sufficient for epileptogenesis in mice harboring the N1768D point mutation in an *Scn8a* allele (“D/+”), their hyperactivity is not required for the tonic phase or concomitant apnea. We utilize mice heterozygous for a patient-derived, gain-of-function *Scn8a* mutated allele that have spontaneous, audiogenic, and hippocampal (HC) stimulated seizures. We found that in spontaneous and HC stimulated seizures, where the focus is likely the temporal lobe, cortical ictal activity is initiated prior to seizure behavior, including wild running and tonic phase apnea. However, in audiogenic seizures cortical ictal activity is not initiated until after tonic phase apnea has begun, suggesting that cortical activity does not drive tonic contraction or apnea. To directly test this hypothesis, we used chemogenetics to selectively inhibit forebrain excitatory neurons during both audiogenic and HC stimulated seizures of D/+ mice. Inhibition of forebrain excitatory neurons was sufficient to suppress electrocorticogram (ECoG) ictal activity in the motor cortex but had no effect on the tonic phase or seizure-induced apnea.

## Materials and methods

### Mouse husbandry and genotyping

All mice were house and cared for in accordance with the Animal Care and Use Committee standards of the University of Virginia. Mice were housed in a temperature and humidity-controlled vivarium with a standard 12-h light/dark cycle with food and water *ad libitum* in accordance with NIH guidelines. Mice harboring the N1768D point mutation in an *Scn8a* allele (“D/+ mice”) were used as a model of epilepsy. To test the role of forebrain excitatory neurons in generation of seizure-induced apnea, we used *Emx1*-Cre mice (Jax # 005628) to genetically target these neurons. To confirm *Emx1*-Cre mice generated recombination specifically in the forebrain (Figure 2), we crossed them with Ai9 reporter mice (Jax # 007909), that express TdTomato from the Rosa26 locus upon Cre mediate recombination (Madisen et al., 2010). To test the efficacy of neuronal inhibition by the synthetic ligand Clozapine-*N*-Oxide (CNO; Figure 3), we crossed *Emx1*-Cre mice with LSL-GiDREADD mice (Jax # 026219), that express GiDREADD receptors from the Rosa26 locus upon Cre mediate recombination (Zhu et al., 2016). The mice for experiments testing the role of forebrain excitatory neurons in seizure behaviors (Figures 4, 5), D/+ mice were crossed with *Emx1*-Cre and floxed-GiDREADD mice. Genotyping was performed by Transnetyx, Inc. (Cordova, TN, United States), with methods of each mouse line as previously described (Wagnon et al., 2015; Bunton-Stasyshyn et al., 2019; Wengert et al., 2021a).

### Surgical preparation

Custom electrocorticogram (ECoG)/electrocardiogram (ECG) headsets (P1 Technologies, Roanoke, VA, United States) were implanted in 6–8-week-old mice using standard aseptic surgical techniques as done previously (Wengert et al., 2021a,b; Wenker et al., 2021, 2022). Anesthesia was induced with 5% and maintained with 0.5–3% isoflurane. Adequacy of anesthesia was assessed by lack of toe-pinch reflex. A midline skin incision was made over the skull and the skull was cleared from connective tissue with 3% peroxide. For mice destined for audiogenic seizures, burr holes were drilled in both the left and right frontal bones to place ECoG leads in the motor cortex at the approximate coordinates of 1 mm rostral, 1 mm lateral, and 1.5 mm ventral to bregma. Bur holes were also drilled in the occipital bone for reference and ground electrodes. Surgery was the same for mice used for HC electrically stimulated seizures, except that a bipolar electrode was implanted into the left hippocampus at coordinates 2 mm caudal, 2 mm lateral, and 2 mm ventral of bregma. The headsets were attached to the skull with dental acrylic (Jet Acrylic; Lang Dental, Wheeling, IL, United States). Two ECG leads were passed subcutaneously to the left abdomen and right shoulder and sutured into place to approximate a lead II arrangement. Mice received post-operative analgesia with ketoprofen (5 mg/kg, i.p.) and 0.9% saline (0.5 ml i.p.) and were allowed to recover a minimum of 5 days prior to experiments.

### Recording of electrocorticogram, electrocardiogram, and breathing

Recording of ECoG, ECG, and breathing was performed as previously described (Wengert et al., 2021b; Wenker et al., 2021). Plethysmography chambers were built to comply with requirements for continuous housing described in the Guide for the Care and Use of Laboratory Animals (Council, 2011). The floor of the chambers had approximate dimensions of 4.5 × 4.5 inches (>20 sq. inches) and 7 inches tall. There were ports for air in and air out, and for pressure monitoring. The chamber was supplied with a continuous flow of room air at approximately 400 ml/min *via* supply and exhaust air pumps (MK-1504 Aquarium Air Pump; AQUA Culture) balanced to maintain chamber pressure near atmospheric. Mice had access to a continuous supply of water and food. The surgically implanted headsets were attached to a custom low torque swivel cable, allowing mice to move freely in the chamber. To assess breathing frequency, the pressure of the EMU chamber was measured with an analog pressure transducer (SDP1000-L05; Sensirion, Stafa, Switzerland). ECoG and ECG signals were amplified at 2000 and bandpass filtered between 0.3–100 and 30–300 Hz, respectively, with an analog amplifier (Neurodata Model 12, Grass Instruments Co., West Warwick, RI, United States). Biosignals were digitized with a Powerlab 16/35 and recorded using LabChart 7 software (AD Instruments, Sydney, NSW, Australia) at 1 kS/s. Video acquisition was performed by multiplexing four miniature night vision-enabled cameras and then digitizing the video feed with a Dazzle Video Capture Device (Corel, Inc., Ottawa, ON, Canada) and recording at 30 fps with LabChart 7 software in tandem with biosignals.

### Seizure recording and stimulation

Spontaneous, audiogenic, and HC stimulated seizures were recorded from separate sets of mice.

Three 8–12 week-old D/+ mice were used for chronic recording of spontaneous seizures. The mice were housed in the plethysmography chambers and recorded as described above 24 h a day and provided with water and food *ad libitum* as we have previous described (Wengert et al., 2021b; Wenker et al., 2021).

**FIGURE 1:**
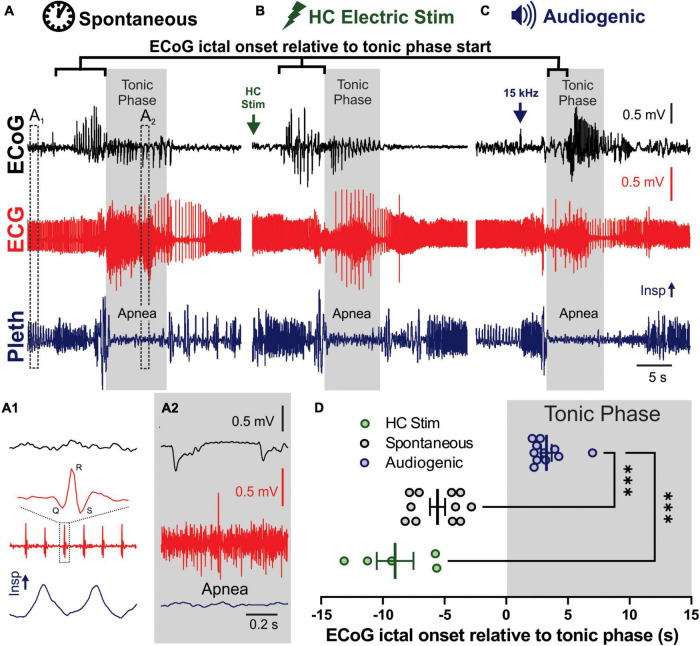
Spontaneous, audiogenic, and hippocampal (HC) electrical stimulated seizures in D/+ mice have similar semiology. **(A–C)** Electrocorticogram (ECoG), electrocardiogram (ECG), and breathing (Pleth) were recorded during spontaneous **(A)**, audio induced **(B)**, and HC electrically stimulated **(C)** seizures. Arrows indicates onset of electrical stimulation **(B)** and 15 kHz acoustic stimulation **(C)**. Gray boxes indicated timing of the tonic phase based on gross electromyography (EMG) activity detected in the ECG signal. (A1) Expanded traces of pre-ictal biosignals from A1 in panel (A) that demonstrates individual QRS complexes in the ECG and inspirations in the pleth. (A2) Same signals during the tonic phase, demonstrating EMG noise in ECG and apnea. **(D)** Audiogenic seizures (blue, *n* = 11 seizures recorded from 5 mice), but not HC stimulated (green, *n* = 5 seizures recorded from 3 mice), showed a delayed onset of ECoG ictal activity relative to tonic phase compared to spontaneous seizures (black, *n* = 8 seizures recorded from 3 mice; ^***^ indicates *p* < 0.001, Dunnett’s multiple comparison test after significant Kruskal–Wallis, KW statistic = 20.27, *p* < 0.0001).

All stimulated seizures were performed between 10 a.m. and 3 p.m. and mice were allowed at least two recovery days in-between seizure stimulations. All spontaneous and stimulated seizures were behaviorally and electrographically tonic seizures, as previously described for spontaneous seizures in these mice (Wengert et al., 2021b; Wenker et al., 2021), and as depicted in Figure 1.

**FIGURE 2:**
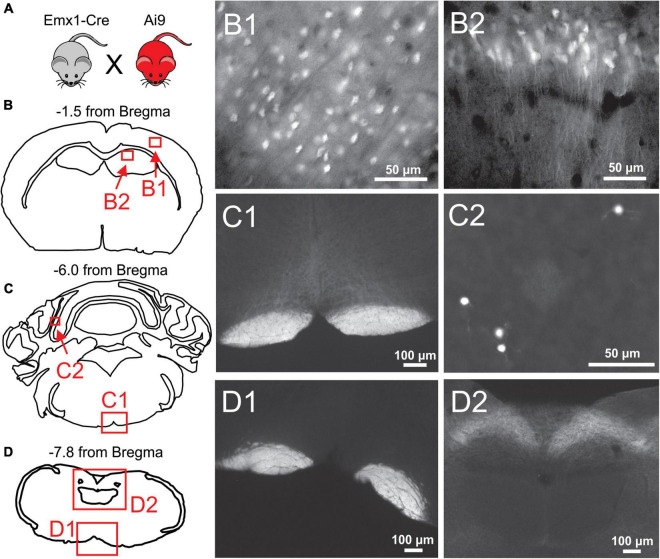
Histological examination of *Emx1*-Cre expression in the cortex and brainstem. **(A)** *Emx1*-Cre mice (Jax #005628) were crossed with a Cre-inducible TdTomato mouse line (Ai9 mice; Jax 007909) to produce Ai9^*Emx*1–*Cre*^. **(B–D)** Locations of fluorescent images taken from transverse brain sections of an Ai9^*Emx*1–*Cre*^ mouse. Recombination, as indicated by TdTomato fluorescence in cell bodies, was observed in Pyramidal cells of Layers 2/3 of the cortex (B1), and the cell layers of the hippocampus, including the CA1 (B2) and dentate gyrus (data not shown). No cell somata were observed in any brainstem sections analyzed (12 analyzed in total, 3 depicted here as examples). As expected, the pyramidal tracts expressed TdTomato in all brainstem sections (examples in panels C1,D1), as these are axons of passage originating from pyramidal neurons. Sparse recombination was observed in cell bodies of the cerebellar granule layer (C2). In addition, what appeared to be terminal fields or diffuse axonal processes routinely were in the nucleus of the solitary tract (NTS; D2).

**FIGURE 3:**
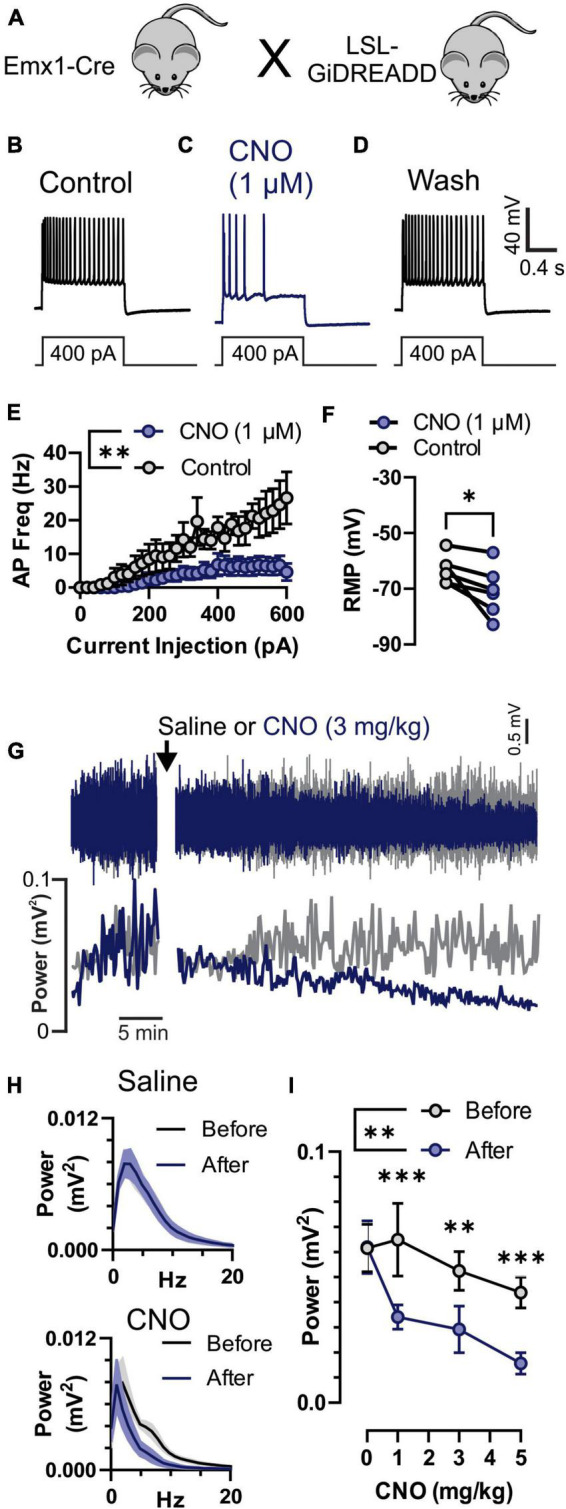
Inhibition of neuronal activity by Clozapine-*N*-Oxide (CNO) in GiDREADD^*Emx*1–*Cre*^. **(A)** *Emx1*-Cre mice were crossed with a Cre-inducible Gi-DREADD receptor mouse line to produce GiDREADD^*Emx*1–*Cre*^ mice. **(B–D)** Current clamp recordings from a layer 5 pyramidal neuron during a 1 s 400 pA square current pulse. Addition of 1 μM CNO to the bath solution resulted in hyperpolarization of the resting membrane potential (RMP) and fewer action potentials (APs) upon current inject (panel **C** compared to panels **B** or **D**). **(E)** APs generated by increasing current pulses from 0 to 600 pA were detectably less in the presence of 1 μM CNO compared to control (*n* = 5 cells, 3 mice). **(F)** RMP in 1 μM CNO and control (*n* = 5 cells, 3 mice). **(G)** Raw electrocorticogram (ECoG) signal and spectral power (0.5–20 Hz) from motor cortex before and after i.p. injection of saline (gray trace) or 3 mg/kg CNO (blue trace). **(H)** Average ECoG power spectrum histograms (*n* = 5 mice) for before (black) and 30–45 min after (blue) saline (top) and 3 mg/kg CNO (bottom) treatments. **(I)** Average ECoG spectral power (0.5–20 Hz; *n* = 5 mice) before (gray) and 30–45 min after (blue) i.p. injection of 0, 1, 3, and 5 mg/kg CNO. CNO reduced ECoG power at all doses. *, ^**^, and ^***^ indicate *P* < 0.05, 0.01, and 0.001, respectively.

**FIGURE 4:**
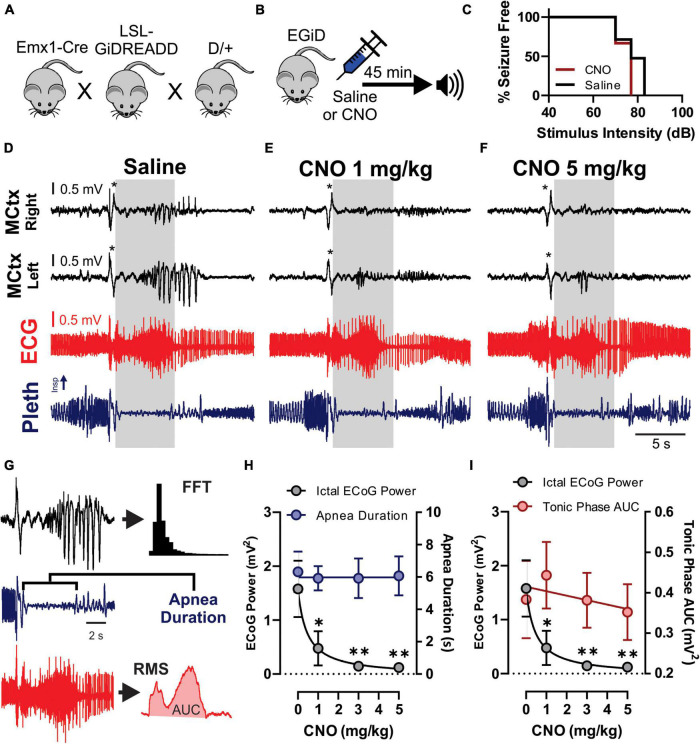
Clozapine-*N*-Oxide (CNO) suppresses cortical ictal activity but not apnea associated with audiogenic seizures. **(A)** D/+ mice were crossed with *Emx1*-Cre and floxed-GiDREADD mice to produce D/+ mice engineered with GiDREADD receptors expressed in cortical neurons (EGiD mice). **(B)** Depiction of experimental paradigm where EGiD mice were injected with saline or CNO 45 min prior to stimulation of an audiogenic seizure. **(C)** EGiD mice were exposed to increasing sound intensities until seizures were initiated. There was no detectable change in audiogenic seizure threshold between control and CNO (5 mg/kg) conditions. (D–F) Right and left motor cortex electrocorticogram (ECoG) activity (MCtx), electrocardiogram (ECG), and breathing (pleth) were recorded during audiogenic seizures after injection with saline **(D)**, 1 mg/kg CNO **(E)**, and 5 mg/kg CNO **(F)**. Asterisks denote movement artifact from brief wild running phase that routinely occurs prior to tonic and clonic phases in D/+ mice after audiogenic stimulation (Wengert et al., 2021b). Gray boxes represent the tonic phase. Note: cortical spike wave discharges that occur during seizures are blocked by CNO but not by saline. **(G)** Depiction of analysis of ECoG power (black), apnea duration (blue), and tonic phase area under the curve (AUC, red). **(H,I)** Plots of ictal ECoG power (gray) vs. apnea duration (**G**; blue; *n* = 7 mice) and tonic phase AUC (**H**; red; *n* = 6 mice). * and ^**^ and *p* < 0.05 and 0.01, respectively.

**FIGURE 5:**
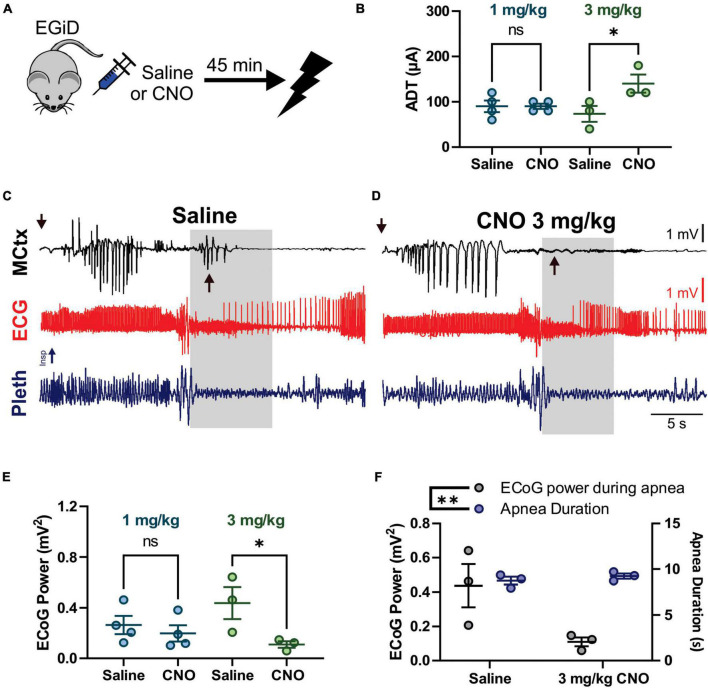
Clozapine-*N*-Oxide (CNO) suppresses cortical ictal activity during the tonic phase, increases after-discharge threshold (ADT) levels, but does not affect apnea or early seizure cortical ictal activity for hippocampal (HC) stimulated seizures. **(A)** Depiction of experimental paradigm where EGiD mice were injected with saline or CNO 45 min prior to HC stimulated seizure. **(B)** ADT comparing saline to 1 mg/kg CNO (aqua) and 3 mg/kg CNO (green). **(C,D)** Left motor cortex electrocorticogram (ECoG) activity (MCtx), electrocardiogram (ECG), and breathing (pleth) were recorded during HC stimulated seizures after i.p. injection with saline **(C)** and 3 mg/kg CNO **(D)**. Gray boxes represent the tonic phase. Downward arrows indicate end of HC stimulation. Upward arrows indicate cortical spike wave discharges that occur during the tonic phase. **(E)** Plot of ECoG Power during the tonic phase comparing saline to 1 mg/kg CNO (aqua) and 3 mg/kg CNO (green). **(F)** Plot of ECoG Power (gray) and Apnea Duration (blue) for saline and 3 mg/kg CNO conditions. * and ^**^ and *p* < 0.05 and 0.01, respectively.

Seven 8–10 week-old D/+ mice were used to induce audiogenic seizures at 15 kHz signal pure tone (∼90 dB) generated using Tone Generator software (NCH Software, Inc., Canberra, ACT, Australia), amplified using a Kinter K3118 stereo amplifier (Kinter, Waukegan, IL, United States), and converted to sound using a small 3-watt speaker lowered into the plethysmography chamber. To determine the effect of CNO administration on audiogenic seizure threshold, a subset of four mice were used. On separate days mice were either injected with saline or 5 mg/kg CNO, placed in a cage, and exposed to increasing intensities (48–95 dB) of a 15 kHz tone for 30 s.

Five 8–10-week-old D/+ mice were used for HC stimulated seizures. Mice were placed in the chamber 30–60 min prior to stimulation. Stimulation of seizures was achieved by connecting the two HC leads to an isolated pulse stimulator (Model 2100, A-M Systems, Inc., Sequim, WA, United States) and stimulating 2 s trains of 1 ms biphasic current pulses at 50 Hz. Stimulations were repeated every 60 s with increased current amplitude (20–600 μA, in 20 μA increments) until a seizure was produced. The final current amplitude that produced a seizure was recorded as the after-discharge threshold (ADT) for that stimulation.

### *In vivo* biosignal analysis

*In vivo* electrophysiological data was analyzed with Spike2 software (Cambridge Electronic Design, Ltd, Cambridge, United Kingdom). Determination of cortical ictal activity and tonic phase timing (Figure 1), apnea duration (Figures 4, 5), and ECoG power (Figures 3–5) were done by an experimenter blinded to the treatment at the time of analysis. The beginning of cortical ictal activity was defined as the time of the first spike wave discharge that was not due to the movement artifact commonly observed during wild running immediately before the tonic phase. The beginning of the tonic phase was defined as the time when tonic muscle electrical activity first became apparent during the seizure, and was confirmed in the video as the time when hindlimb extension began. Apnea duration was measured as the time difference between the two detectable breaths on either side of the apnea; breaths were detected based on a rising threshold set by the experimenter that was sufficient to detect all normal breaths preceding the seizure by 30 s, as we have done before (Wengert et al., 2021b; Wenker et al., 2021). ECoG power was performed with Spike2 software’s built in Fast Fourier Transformation (FFT) using a Hanning window with a window size of 1024 data points (1.024 s), resulting in a frequency resolution of 0.9766 Hz. Within an individual mouse, the same amount of time was analyzed for seizures under each condition (i.e., saline and CNO dosages). Tonic phase area under the curve (AUC) was also calculated in Spike2 by first deriving root mean square (RMS) of the ECG signal and then measuring the AUC between the beginning and end of the tonic phase (Figure 4F, middle). Reported ECoG power and tonic phase AUC was always expressed as mV^2^.

### *In vitro* patch clamp electrophysiological recording

Preparation of acute brain slices for patch-clamp electrophysiology experiments was modified from standard protocols previously described (Wengert et al., 2021a). Mice were anesthetized with isoflurane and decapitated. The brains were rapidly removed and kept in chilled Artificial Cerebrospinal Fluid (ACSF) (0°C) containing the following (in mM): 125 NaCl, 2.5 KCl, 1.25 NaH_2_PO_4_, 2 CaCl_2_, 1 MgCl_2_, 0.5 L-ascorbic acid, 10 glucose, 25 NaHCO_3_, and 2 Na-pyruvate (osmolarity 310 mOsm). The slices were continuously oxygenated with 95% O_2_ and 5% CO_2_ throughout the preparation; 300 μm coronal or horizontal brain sections were prepared using a Leica Microsystems VT1200 vibratome. Slices were collected and placed in ACSF warmed to 37°C for ∼30 min and then kept at room temperature for up to 6 h.

Brain slices were placed in a chamber continuously superfused (∼2 ml/min) with continuously oxygenated recording solution. Layer 5 pyramidal neurons were identified based on anatomical location and pyramidal morphology *via* DIC video microscopy using a Carl Zeiss Axioscope microscope. Whole-cell recordings were performed using a Multiclamp 700B amplifier with signals digitized by a Digidata 1322A digitizer. Currents were amplified, low-pass filtered at 2 kHz, and sampled at 100 kHz. Borosilicate electrodes were fabricated using a Brown-Flaming puller (model P1000, Sutter Instruments, Novato, CA, United States) and had pipette resistances between 1.5 and 3.5 mΩ.

Current-clamp recordings of neuronal excitability were collected in ACSF solution identical to that used for preparation of brain slices. The internal solution contained the following (in mM): 120 K-gluconate, 10 NaCl, 2 MgCl_2_, 0.5 K_2_EGTA, 10 HEPES, 4 Na_2_ATP, 0.3 NaGTP, pH 7.2 (osmolarity 290 mOsm). Intrinsic excitability was assessed using methods adapted from those previously described (Ottolini et al., 2017; Wengert et al., 2019, 2021a). Action potential (AP) frequency–current relationships were determined using 1 s current injections ranging from 0 to 600 pA. APs were counted as deflections that rose above 0 mV. Resting membrane potential (RMP) was determined from the period prior to current injection. Data was acquired with Clampex software and analyzed with Clampfit software (Molecular Devices, San Jose, CA, United States).

### Pharmacology

Clozapine-*N*-Oxide was purchased from Sigma-Aldrich, St. Louis, MO, United States For *in vivo* experiments, CNO was dissolved into sterile saline at a concentration of 0.5 mg/mL, and was sterile filtered prior to injection. Injections were given intraperitoneal (i.p.) in a volume of 50–250 μl per mg of mouse weight 30–45 min prior to seizure induction by audio or HC stimulation. For *in vitro* experiments, CNO was dissolved into dimethyl sulfoxide (DMSO) at a concentration of 10 mM and diluted 10,000-fold into ACSF for experiments.

### Immunohistochemistry

Mice were deeply anesthetized with phenobarbital and transcardially perfused with 10 ml ice-cold Dulbecco’s PBS (DPBS) followed by 10 ml ice-cold 4% PFA. Brains were removed and immersed in 4% PFA overnight at 4°C. Brains were then washed with DPBS with 0.1% sodium azide at 4°C prior embedding in 2% agarose and making 40 μm transverse sections using a vibratome (Leica Microsystems, Wetzlar, Germany, VT1200). Tissues were mounted on slides using AquaMount (Polysciences, Warrington, PA, United States). Microscopy and imaging were done using the Neurolucida system (MBF Bioscience, Williston, VT, United States) with a Zeiss Axioskop microscope with computer-driven stage and Zeiss MRc camera.

### Statistical analysis

All data points in Figures 3I, 4G, H, 5B, E, F denote biological replicates (i.e., no animal was used more than once for the same test). Data comparing spontaneous and electrically stimulated seizures (Figure 1) and data comparing in current clamp neuronal responses to CNO (Figure 3) are technical replicates and the animal numbers are reported in the figure legends. All average data values are expressed as mean ± SEM. Descriptive statistics and statistical tests were computed using GraphPad Prism version 9 (GraphPad Software, Inc., San Diego, CA, United States), except for MANOVAs, which were computed using SPSS (IBM, Armonk, NY, United States). Comparisons were considered statistically detectable when *P* < 0.05. All data was evaluated for normality of residuals by the Shapiro–Wilk test, and the appropriate non-parametric test was used when residuals were not normal.

## Results

### Comparison of spontaneous, audiogenic, and hippocampal stimulated seizures in D/+ mice

Spontaneous (Figure 1A), HC electrically stimulated (Figure 1B), and audiogenic (Figure 1C) seizures of D/+ mice all have very similar semiology (Supplementary Video 1), including brief wild running prior to a pronounced tonic phase coincident with apnea (Wengert et al., 2021b; Wenker et al., 2021, 2022). One difference stands out: all recorded spontaneous and HC stimulated seizures produced cortical ictal activity that preceded the tonic phase; however, cortical ictal activity was delayed until after initiation of the tonic phase of audiogenic seizures (Figures 1C,D), suggesting that cortical ictal activity is not necessary for production of the tonic phase or apnea. To further investigate this we crossed the D/+ mice with mice homozygous for *Emx1*-Cre and LSL-GiDREADD receptors, to produce mice with GiDREADD receptors expressed in forebrain excitatory neurons of D/+ mice to selectively inhibit forebrain excitatory neurons during both audiogenic and HC stimulated seizures.

### Confirmation of forebrain excitatory neuronal inhibition by Clozapine-*N*-Oxide

To verify whether Cre-mediated recombination of *Emx1*-Cre mice is restricted to forebrain excitatory neurons, *Emx1*-Cre mice were crossed with Ai9 mice to produce Ai9^*Emx*1–*Cre*^ mice (Figure 2A), resulting in TdTomato production in cells that express Cre. As previously reported (Iwasato et al., 2000; Gorski et al., 2002; Madisen et al., 2012), many neurons were fluorescently labeled in the forebrain of Ai9^*Emx*1–*Cre*^ mice (Figure 2B) and no cell bodies were observed in the brainstem. In the brainstem, labeling was exclusively found in neuronal fibers and not in cell bodies (Figures 2C,D), with the exception of sparse labeling of somata in the cerebellum (Figure 2C2). As expected, neuronal fibers were mostly restricted to the pyramidal tract, a major output of excitatory neurons of the motor cortex. Fiber tracts with robust labeling were also found in the tractus solitarius and its terminal fields in the nucleus of the solitary tract, the primary nucleus for visceral sensory relays (Llewellyn-Smith and Verberne, 2011; Figure 2D2).

To confirm that CNO does inhibit cortical neuronal activity we recorded individual pyramidal neurons in the motor cortex *in vitro* and motor cortex ECoG *in vivo* in *Emx1*-Cre mice crossed with LSL-GiDREADD mice to produce GiDREADD^*Emx*1–*Cre*^ (Figure 3A). We performed whole-cell current-clamp recordings from layer V pyramidal neurons that were identified by their anatomical location, shape, and firing properties (Wengert et al., 2019, 2021a). Depolarizing current injection steps resulted in generation of APs (Figure 3B) that were reversibly inhibited by application of 1 μM CNO (Figures 3C,D). CNO (1 μM) detectably reduced AP firing across a range of current pulse amplitudes [Figure 3E; *p* = 0.0084, *F*_(1,8)_ = 12.08, treatment factor of 2-way ANOVA] and detectably hyperpolarized resting membrane potential (RMP; Figure 3F; *p* = 0.0393, 2-tailed paired *t*-test). We also monitored ECoG activity (Figure 3G, top) and spectral power (Figure 3G, bottom) from the motor cortex in GiDREADD^*Emx*1–*Cre*^ mice before and after i.p. injection with CNO or saline. Spectral power was decreased 30–50 min after injection of CNO across 0.5–20 Hz (Figure 3H, bottom), while saline injection had minimal impact (Figure 3H, top). The decrease in spectral power due to CNO injection was detectable at 1, 3, and 5 mg/kg doses [Figure 3I; *p* = 0.0004, 0.0039, and 0.0008, respectively; after significant treatment factor of 2-Way ANOVA, *p* = 0.0071, *F*_(1,4)_ = 25.79].

### Inhibition of cortical excitatory neurons does not affect tonic phase apnea of audiogenic seizures

To test the hypothesis that excitatory neurons of the cortex do not contribute to tonic muscle contraction and apnea, we used chemogenetics to inhibit cortical neurons while inducing audiogenic seizures. We crossed D/+ mice with *Emx1*-Cre and LSL-GiDREADD mice to produce mice heterozygous for *Emx1*-Cre, LSL-GiDREADD, and the N1768D mutation, which we refer to as EGiD mice (Figure 4A). Seven mice were each tested at four doses of CNO: 0 (i.e., saline control), 1, 3, and 5 mg/kg of CNO (Table 1). Audiogenic seizures were tested ∼45 min after injection (Figure 4B). Mice were always given a minimum of 48 h to recover between trials and the order of CNO dosage administration was random. Even at a high dose of CNO (5 mg/kg), seizure threshold was unchanged (Figure 4C; *p* = 0.6056, Chi-square = 0.2667, Gehan–Breslow–Wilcoxon test). As described above, all audiogenic seizures have apnea coincident with the tonic phase and cortical ictal activity occurs after the tonic phase begins (Figure 4D). Administration of both high (5 mg/kg) and low (1 mg/kg) doses of CNO suppressed cortical ictal activity measured directly from the motor cortex region (Figures 4E,F, black traces). However, tonic muscle contraction and apnea were unchanged (Figures 4E,F, red and blue traces). To quantify this, we assessed ictal ECoG power by FFT (Figure 4G, top), apnea duration as the time between observed inspirations (Figure 4G, middle) and extent of the tonic phase by RMS amplitude (Figure 4G, bottom; time constant = 0.5 s). ECoG power, but not apnea duration, was detectably decreased by 1, 3, and 5 mg/kg doses of CNO compared to control (Figure 4H; *p* = 0.0046, 0.008, and 0.007, respectively for ECoG power; *p* = 0.844, 0.847, and 0.954, respectively for apnea duration; using Dunnett’s multiple comparison after significant MANOVA, Pillai’s Trace = 1.325, *F* = 11.773, *p* > 0.001). Similarly, while ECoG power was detectably decreased by 1, 3, and 5 mg/kg doses of CNO compared to control, tonic phase AUC was not affected (Figure 4I; *p* = 0.0046, 0.008, and 0.007, respectively for ECoG power; *p* = 0.910, 1.000, and 0.985, respectively for apnea duration; using Dunnett’s multiple comparison after significant MANOVA, Pillai’s Trace = 1.205, *F* = 7.583, *p* > 0.001).

**TABLE 1:** Mouse usage for audiogenic and HC stimulated seizures.

Mouse	Seizure type	Saline	1 mg/kg CNO	3 mg/kg CNO	5 mg/kg CNO
EGiD_44	Audiogenic	X	X	X	X
EGiD_117	Audiogenic	X	X	X	X
EGiD_132	Audiogenic	X	X	X	X
EGiD_134	Audiogenic	X	X	X	X
EGiD_143	Audiogenic	X	X	X	X
EGiD_162	Audiogenic	X	X	X	X
EGiD_163	Audiogenic	X	X	X	X
EGiD_178	HC stimulated	X	X	X	No seizure
EGiD_184	HC stimulated	X	X	X	No seizure
EGiD_217	HC stimulated	X	X	No seizure	No seizure
EGiD_216	HC stimulated	X	X	X	No seizure
EGiD_219	HC stimulated	X	Not tested	X	X

“X” indicates seizure was stimulated in mouse at this dose and data as included for analysis.

### Inhibition of cortical excitatory neurons decreases hippocampal stimulated seizure susceptibility, but does not affect tonic phase apnea

We also wanted to test the hypothesis that excitatory neurons of the cortex do not contribute to tonic muscle contraction nor apnea during seizures that originate in the forebrain. To this end, we used HC stimulated seizures in D/+ mice, which have identical seizure semiology to that observed with spontaneous seizures (Figure 1). We injected EGiD mice either with saline or CNO prior to HC stimulation of seizures ∼45 min later (Figure 5A). Mice were always given a minimum of 48 h to recover between trials and the order of CNO dosage administration was random. For every HC stimulated seizure, the ADT was determined by increasing the current pulse amplitude until a seizure occurred. Only mice with successful seizure stimulation with low ADT under saline conditions were included. Five mice met these criteria. Of the five mice, we were able to generate HC stimulated seizures in only one mouse at 5 mg/kg CNO, 3 mice at 3 mg/kg CNO, and 4 mice at 1 mg/kg CNO (Table 1). In the 4 mice tested for HC stimulated seizures in the presence of 1 mg/kg CNO, mean ADT intensities were unchanged [Figure 5B; *p* > 0.9999, Holm–Sidak’s multiple comparison test after significant one-way ANOVA, *p* = 0.0468, *F*_(3,10)_ = 3.810]. At the higher concentration of 3 mg/kg CNO, ADT intensities were detectably elevated [Figure 5B; *p* = 0.0201, Holm–Sidak’s multiple comparison test after significant one-way ANOVA, *p* = 0.0468, *F*_(3,10)_ = 3.810]. Although seizure threshold was increased by 3 mg/kg CNO, the semiology of resultant seizures was unaffected (Figures 5C,D). Routinely, we found that HC stimulated seizure had two periods of cortical ictal activity. The first was initiated during or immediately after the electrical stimulation (downward arrows in Figures 5C,D). The second period of ictal activity occurred during the tonic phase (upward arrows in Figure 5C). The ictal activity that occurred during the tonic phase was detectably inhibited by 3 mg/kg CNO administration (upward arrows in Figure 5D) but not by 1 mg/kg CNO administration [Figure 5E; *p* > 0.9999 and *p* = 0.0201, for 1 and 3 mg/kg CNO, respectively, Sidak’s multiple comparison test after significant one-way ANOVA, *p* = 0.0468, *F*_(3,10)_ = 3.810]. Although cortical ictal activity during the tonic phase was inhibited by 3 mg/kg CNO, apnea duration was unaffected (Figure 5F; MANOVA, Pillai’s Trace = 1.634, *F* = 8.934, *p* = 0.005).

## Discussion

The primary finding of the current study is that synchronous activity of excitatory cortical neurons is not essential for instigation of the tonic phase and concurrent apnea of a seizure. This was true for both seizures that were initiated in the temporal lobe and audiogenic seizures, which are believed to originate in subcortical structures (Faingold, 2012). Thus, the current data suggests that regardless of how a seizure is formed, generation of the tonic phase and seizure-induced apnea is produced by neural circuitry in the mid- and hindbrain.

### *Scn8a* mutant mice as a model of seizure-induced apnea and sudden unexpected death in epilepsy

*SCN8A* epileptic encephalopathy is a severe genetic epilepsy with a high risk of SUDEP (Larsen et al., 2015; Gardella and Møller, 2019). In addition, non-fatal tonic seizures have been witnessed in numerous patients. As we and others have reported, these tonic seizures present with similar semiology to those of the *Scn8a* mutant mouse models: that is, with apnea and generalized breathing dysfunction, in addition to bradycardia (Trivisano et al., 2019; Zawadzka et al., 2020; Wenker et al., 2021). Although cases of SUDEP in *SCN8A* patients have been unwitnessed, tonic seizure and the resultant apnea appear as a likely mechanism of fatality. Indeed, in *Scn8a* mutant mice we have found that tonic seizure and apnea produce the spontaneous death observed in these models (Wengert et al., 2021b; Wenker et al., 2021). As discussed below, tonic seizures are a feature of other models of SUDEP; thus, the *Scn8a* mutant mouse model utilized in this study represents both an ideal clinical and broadly applicable model of SUDEP and seizure-induced apnea.

### The tonic phase and seizure-induced apnea

In the D/+ mice, spontaneous seizures are quite similar in semiology to both audiogenic (Wengert et al., 2021b) and HC stimulated seizures (Wenker et al., 2022). These seizures are of the tonic variety: a brief wild running phase is followed by a prolonged tonic phase, with occasional clonic activity prior to recovery (Wengert et al., 2021b). Tonic seizures appear common in other models of SUDEP (Faingold et al., 2017; Martin et al., 2020) and are associated with apnea in patients (Gastaut et al., 1963; Wyllie, 2015; Zawadzka et al., 2020; Wenker et al., 2021). Thus, the association between the tonic phase and seizure-induced apnea appears to be strong. In mice, there is also an association between the tonic phase and seizure-induced death–i.e., the experimental correlate of SUDEP (Martin et al., 2020; Wengert et al., 2021b; Wenker et al., 2021). Whether this tonic phase apnea occurs in instances of clinical SUDEP is unclear; however, some epilepsies that commonly present with frequent tonic seizures (e.g., tuberous sclerosis complex, Lennox-Gastaut syndrome, and *SCN8A* EE) do also experience higher mortality rates, often from SUDEP (Autry et al., 2009; Gardella and Møller, 2019; Parthasarathy et al., 2021). Thus, we propose the tonic phase and concomitant apnea as a possible mechanism of SUDEP, likely amongst others.

### The role of forebrain excitatory neurons in seizure generation and semiology

We have previously shown that expression of a *Scn8a* gain-of-function mutation selectively in the forebrain is sufficient to produce spontaneous convulsive seizures and seizure-induced death (Bunton-Stasyshyn et al., 2019; Wenker et al., 2021). In the present study, we used mice with a *Scn8a* gain-of-function mutation expressed in the germline and expressed synthetic receptors that activate the Gi pathway (GiDREADD receptors) in excitatory forebrain neurons (Zhu et al., 2016) suppressing neuronal activity. We were able to demonstrate neuronal inhibition at the cellular level and *in vivo* during seizures. Perhaps unsurprisingly, we found that inhibition of excitatory forebrain neurons reduces susceptibility to HC stimulated seizures. However, once initiated, both HC stimulated and audiogenic seizures produced identical semiology: wild running followed by a prolonged tonic phase with apnea, and this was unaffected when cortical neurons were inhibited with CNO. Interestingly, we also observed fiber tracts projecting into the NTS that experienced recombination. It is likely that these peripheral neurons were also inhibited by CNO in our experiments, but this also had no impact on the tonic phase or seizure-induced apnea. Importantly, these results do not affect our conclusion that cortical neurons do not drive the tonic phase and apnea.

### The neural circuitry of tonic phase apnea

**FIGURE 6:**
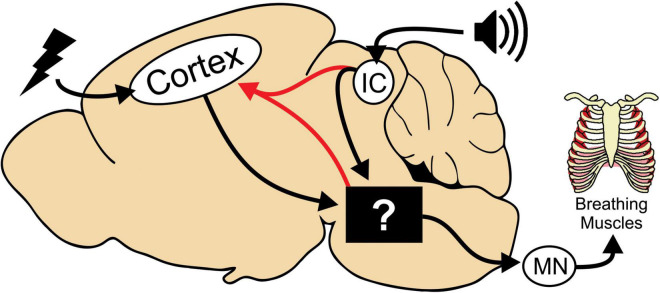
Model depicting neural circuitry involved in tonic phase apnea. In our mouse model, seizures can be initiated in the cortex or in the brainstem *via* the inferior colliculus (IC) for audiogenic seizures. The tonic phase and apnea are likely generated by neural circuitry in the brainstem, whose specific neuronal substrates are yet to be determined. This brainstem circuitry then recruits motor neurons (MN) and can also reactivate cortical ictal activity.

Our interpretation is that although HC stimulated and audiogenic seizures are initiated in different regions of the brain, they impinge on the same circuitry to generate tonic phase apnea (Figure 6, black box). Interestingly, we observed cortical ictal activity during the tonic phase of both HC stimulated and audiogenic seizures. Since this cortical activity occurs with a slight delay to the start of the tonic phase, we propose that it is generated by seizure spread to the cortex from the original seizure focus (e.g., IC) or possibly from the neural circuitry that drives tonic phase apnea (Figure 6, red line). Previous work, while not causal, has implicated the broad region of the brainstem reticular formation in generation of the tonic phase of rodent seizures (Faingold and Randall, 1995; Faingold, 2012). In addition, numerous subcortical regions not depicted in our model could be critical for apnea generation. For instance, the amygdala is strongly implicated in apneas of pediatric epilepsies (Dlouhy et al., 2015; Nobis et al., 2019; Rhone et al., 2020). While these apneas appear different than those we observe, most notably that they do not occur during the tonic phase, the amygdala and other regions could play an important role in seizure-induced apnea.

These ideas are not contradictory to our current study. In fact, our work could provide a framework for future studies using GiDREADD receptors to selectively inhibit different subcortical regions that may contribute to tonic phase apnea. For example, rhombomere-specific Cre mice have been produced that can target different regions of the mid- and hindbrain (Sun et al., 2017). Indeed, when excitatory Designer Receptors Exclusively Activated by Designer Drugs (DREADD) receptors were expressed under control of Cre in these mice, CNO administration produced apnea and death, similar to what seizure-induced death in our mouse models (Wengert et al., 2021b; Wenker et al., 2021, 2022).

## Conclusion

Our findings suggest that while seizures may be generated in the forebrain, the detrimental behaviors (i.e., pathological motor activity and apnea) are generated in the mid- or hindbrain. Understanding the neural circuitry that generates these behaviors has translational value, as they could be targeted with the advancing genetic therapies, such as ASOs (Lenk et al., 2020; Hill and Meisler, 2021; Wengert et al., 2022). Future experiments examining brainstem circuitry that controls respiratory muscles could help us understand and treat potentially fatal seizures.

## Data availability statement

The raw data supporting the conclusions of this article will be made available by the authors, without undue reservation.

## Ethics statement

The animal study was reviewed and approved by University of Virginia Institutional Office of the Vice President for Research Animal Care and Use Committee.

## Author contributions

IW and MP secured the funding, designed the experiments, and co-wrote the manuscript. AB, CL, AT, RM, JH, and PS the performed experiments and analyzed the results. All authors contributed to the article and approved the submitted version.

## Supplementary material

Supplementary Video 1 Side-by-side video of spontaneous, audiogenic, and hippocampal (HC) stimulated seizures in D/+ mice. Text indicates approximate timing of wild running, tonic phase, and recovery periods of the seizures.
